# Method of Nursing Interventions to Reduce the Incidence of Bullying and Its Impact on Students in School: A Scoping Review

**DOI:** 10.3390/healthcare10101835

**Published:** 2022-09-22

**Authors:** Iyus Yosep, Rohman Hikmat, Ai Mardhiyah, Helmy Hazmi, Taty Hernawaty

**Affiliations:** 1 Department of Mental Health, Faculty of Nursing, Universitas Padjadjaran, Bandung 40132, Indonesia; 2 Faculty of Nursing, Universitas Padjadjaran, Bandung 40132, Indonesia; 3 Department of Pediatric Nursing, Faculty of Nursing, Universitas Padjadjaran, Bandung 40132, Indonesia; 4 Department of Nursing, Faculty of Medicine, University of Malaysia Sarawak, Kota Samarahan 94300, Malaysia

**Keywords:** bullying, school, students

## Abstract

The bullying of students in schools can have a negative impact on students. The impact of bullying can take the form of anxiety, low psychological well-being, low social adjustment, psychological distress, and risk of suicide. Incidents of bullying are still considered normal, and there has not been a focus on reducing their incidence and impact. The purpose of this study is to describe nursing interventions to reduce the incidence of bullying and its impact on students at school. This study used the scoping review method and literature reviews via CINAHL, PubMed, and ProQuest databases. The keywords used in English were “bullying OR cyberbullying OR aggression” AND “students OR school students” AND “school OR schools” AND “nursing intervention”. The criteria for articles in this study were: full texts, student populations and samples, randomized control trials or quasi-experiment research designs, use of the English language, and the publication period of the last 10 years (2013–2022). We found 11 articles discussing nursing interventions to reduce the incidence of bullying and its impact on students in schools. There are four types of interventions that can be provided to students, namely, prevention programs, activities programs, peer group programs, and resilience programs. Most of the articles used randomized control trials and quasi-experiment designs. The samples in the articles analyzed were in the range of 50–7121 students. These four types of interventions can reduce the incidence of bullying and its impact on students at school, and in addition, they can improve the social skills and mental health of students, for example, by increasing their self-efficacy, resilience, and adaptive coping. There are four nursing interventions to reduce the incidence of bullying and its impact on students in schools, namely, the prevention program, activities program, peer group program, and resilience program.

## 1. Introduction

The phenomenon of bullying is something that often happens everywhere, including in the school environment. Data from the United Nations Children’s Fund (UNICEF) (2017) show that out of 100,000 children in 18 countries, 67% of children have experienced bullying: 25% of children were bullied because of their physical appearance, 25% because of their gender, 25% because of their ethnicity or gender [1] or their country of origin, and 25% for other reasons. In America, there are as many as 15,600 students from elementary to high school: 17% of them reported being victims of bullying, and 19% admitted that bullying occurred in the school environment [2]. Meanwhile, in Indonesia, as many as 50% of teenagers have experienced bullying [1]. The large number of victims of bullying is caused by several factors in the school environment.

The factors that can cause bullying, namely, family, peer, and school factors, can also shape bullying behavior in adolescents; when these three factors are not conducive, adolescents will tend to vent their emotional turmoil in negative ways, and one of them is bullying [3,4]. Bullying occurs due to several factors, namely, economic differences, religion, gender, traditions, and the habit of seniors to punish their juniors which often occurs [5]. There was a feeling of revenge or envy and a desire to dominate the victim using physical or sexual force [6,7]. In addition, perpetrators bully others to increase their popularity among their peers (peer groups) [8]. These various bullying factors have an impact on the victims of bullying behavior.

The impact experienced by victims of bullying includes sleep disturbances, headaches, stomach pains, depression, and bedwetting [2,9]; emotional and behavioral disorders [9,10], anxiety disorders [11], somatic disorders [12]; low psychological well-being, low social adjustment ability, and psychological distress [13]. In addition, bullying can cause a lack of motivation or self-esteem, mental health problems, nightmares, and a sense of fear. Not infrequently, acts of violence against children also lead to the occurrence of death in the victim [7]. These psychological problems lead to the poor social adjustment of victims of bullying [14,15]. Bullying can also disrupt the psychological growth and development of children as victims can develop tough personalities in the future [16,17,18]. Bullying behavior has a long-term impact, namely, difficulties in socializing, and if this lasts into adulthood, it can have a very broad impact, with victims even experiencing problems with regards to social relations, deteriorating economic conditions, and low welfare at the age of 50 years [18]. Given the serious impact of bullying, it is necessary to prevent bullying-related cases.

Efforts to prevent bullying cases requires the collaboration of various parties, including the Government, teachers, health workers, parents, the role of health workers as counselors, the role of the community environment, and the role of the child himself [19,20]. One program being implemented is in the form of character education, although it is still not running optimally because it does not explain, in detail, issues related to child welfare or violence against children, especially bullying [21]. Another attempt is the provision of education by health workers and teachers regarding preventive measures and ways to overcome the trauma of bullying [22].

Several studies have shown that efforts to reduce bullying behavior can be provided through empathy and mindfulness therapy [23,24]. Subsequent research shows that empathy is effective in reducing bullying behavior in elementary school-age students. Another attempt to reduce bullying behavior is cognitive and affective therapy and from empathy in the counseling process [12].

The impact of bullying behavior on students has both short- and long-term effects. At its worse, it can lead to suicidal thoughts. Therefore, we need interventions that can be implemented to reduce bullying behavior in schools. Interventions to overcome the impact of bullying still focus on the role of schools but have not yet involved the role of health workers. Nurses have not played much of a role in providing interventions to reduce the incidence of bullying and its impact on students at school. Therefore, we need a scoping review that can describe nursing intervention methods to reduce the incidence of bullying and its impact on students in schools so that nurses and schools have a basis for making policies concerning the provision of nursing interventions to reduce the incidence of bullying and its impact on students at school. Therefore, the researchers intend to conduct a literature review with a design scoping review to describe interventions that can be utilized to reduce bullying behavior in students.

## 2. Materials and Methods

### 2.1. Design

The design of this study uses a literature review with a design scoping review. A scoping review is a methodological technique that aims to discuss new topics that are relevant to current events [25]. This research framework is able to achieve the research objectives because it can discuss various studies with a wide conceptual range [26]. The framework in this research consists of 5 core stages, namely, identification of research questions, identification of relevant study results, study selection, data mapping, compilation of results, and reporting of study results [27]. This literature review uses the PRISMA Extension for Scoping Reviews (PRISMA-ScR) to identify various topics that address interventions to reduce bullying behavior in students in schools.

### 2.2. Search Methods

To search for publications, 3 databases were used, namely: PubMed, CINAHL, and ProQuest. The keywords used were: “bullying OR cyberbullying OR aggression” AND “students OR school students” AND “school OR schools” AND “nursing intervention”. The research question was: How are interventions to reduce the impact and behavior of bullying on students at school?

### 2.3. Inclusion and Exclusion Criteria

**Figure 1:**
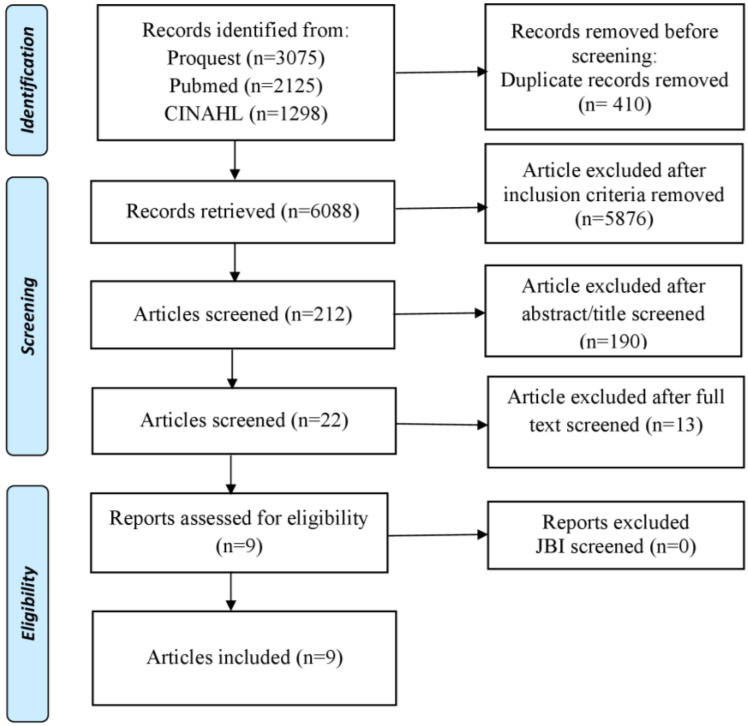
PRISMA flow diagram.

This literature review uses the PRISMA Extension for Scoping Review (PRISM -ScR) which serves to identify various topics that discuss interventions to reduce the impact and behavior of bullying on students in schools (Figure 1). Articles were selected for review based on inclusion and exclusion criteria. The inclusion criteria of this study were: the patient was a student; it was primary research; there was an intervention; the article was original research; and the article was a full text in English, published within the last 10 years (2013–2022).

### 2.4. Data Extraction

After the article was read by all authors, the author composed a summary and an analysis of the results to be entered into the extraction table. The articles were manually extracted using a table containing the author, year, country, study design, population and sample, procedures, interventions, and results of the study. The purpose of creating this data extraction table was to make it easier for the author to describe the results of the review.

### 2.5. Quality Appraisal

Journals were analyzed using the Joanna Briggs Institute (JBI) critical assessment method with good article standards above 75% based on criteria and topic relevance. JBI critical assessment is a critical tool in the assessment of the quality, trustworthiness, and relevance of published papers. The assessment criteria are attributed a score of “yes”, “no”, “unclear”, and “not applicable”, and each criterion with a “yes” score is awarded 1 point, while the other answers are awarded 0 points. Each point of the score is summed to determine the eligibility of the article. The standard of eligible articles was a score above 75% according to the relevance of the topic and the criteria for the articles assessed.

### 2.6. Data Analysis

Then the full text of the articles collected were read by all authors. The authors used a descriptive analysis method. After reading the full texts, the author analyzed the articles in depth, and then they were composed into the form of manual tables. The author then wrote a description of the results of the scoping review to be discussed with previous studies. After being analyzed, various interventions were obtained, classified based on similar interventions, and finally described in the discussion.

## 3. Results

The number of articles obtained from the search was 5407 articles. After checking for duplication in the collected articles, 4985 articles were obtained. Furthermore, after elimination based on the inclusion criteria, there were 4806 articles left. Then after checking the title and abstract, 11 articles were remaining. Articles were analyzed using the JBI critical appraisal tool assessment method with good article standards above 75% based on criteria and topic relevance (Table 1).

We found 11 articles that discuss nursing interventions to reduce the incidence of bullying and its impact on students at school. From the 11 articles, 10 interventions were obtained. We classified them into four types of interventions and then described the results of the study.

### 3.1. Prevention Program

There are three ways to reduce bullying through preventive measures [20]. First, a social cognitive theory (SCT)-based intervention can be implemented in the effort to reduce bullying. The nursing intervention was carried out over four training sessions over six weeks for students. In addition to students, training was also given to teachers and parents. The six components of the intervention were education about bullying, self-efficacy in controlling bullying, social support, norms, expectations, and building perceptions. The SCT-based intervention was proven to be effective in reducing bullying and victimization as well as increasing social competence so as to reduce the bullying behavior in students at school.

Second, preventive efforts to overcome bullying can be carried out through the Bullying Prevention Program (BPP) [28]. This intervention was carried out for one year, and training was provided within 10 × 90 min of lessons per month. The preventive measures included education through film clips, group discussions, and exercises. In addition, online game content that could be played at school and at home, posters on the school buildings, and vests for a campaign to prevent bullying were added. The BPP intervention showed no significant effect on reducing bullying behavior, but it improved the social skills of students.

Third, preventive measures can be implemented through a pragmatic school-based universal intervention [30]. This intervention was designed to address the internal factors that cause bullying including cooperation/communication, empathy, goals/aspirations, problem-solving, self-awareness, and self-efficacy. In addition, this intervention also addressed external factors, namely, school support and caring peer relationships. The intervention was carried out by providing education and training to students and teachers in school. This intervention showed no significant effect on the incidence of bullying but improved the mental health of students at school.

### 3.2. Activities Program

The activity-based program consisted of two activities, namely, in the form of sports and art. Sports activities, namely, Shotokan Karate, were carried out in school for 12 × 60 min, once per week for 12 weeks [31]. Each session was led by a sports expert for the karate training and by a psychologist for the psychosocial intervention. After the intervention, students experienced significantly increased resilience and well-being, and participants did not engage in aggressive or bullying behavior.

The martial arts-based intervention was conducted face-to-face in school [33]. The intervention was carried out for 10 × 50 min, once per week for 10 weeks. The interventions carried out were psychoeducation guided by the facilitator, warm-up activities, stretching activities, Pencak Silat practice techniques, pattern exercises, debates, and meditation focused on breathing exercises. Martial arts-based interventions can increase resilience and self-efficacy.

### 3.3. Peer Group Program

The peer group program consisted of two interventions, namely, the Tutoría Entre Iguales Program (TEI) or school-based intervention of peer tutoring and the Learning Together intervention (LTI). The TEI intervention was carried out with peer guidance oriented towards violence in schools and cyberbullying [32]. This intervention was aimed at improving the school climate and promoting positive schools as well as implementing a culture of tolerance. This intervention was carried out through the collaboration of various elements in the school. The results of the intervention showed a decrease in bullying, peer victimization, fighting, and cyberbullying. In addition, this intervention also promoted a positive school climate.

The next peer group intervention that can be utilized is the Learning Together intervention (LTI) [34]. This intervention was carried out by all staff. Students were given a manual, and meetings were held twice per semester. Discussions on bullying, social and emotional skills, and school management were guided by an external facilitator in each group of six students. Students were also facilitated to work together during the year of the learning process. The LTI intervention showed little effect on aggressive behavior but displayed a significant effect on bullying behavior. The intervention also showed a positive effect on the school environment by reducing bullying behavior.

### 3.4. Resilience Program

The resilience program included four Internet-based interventions including Cultivating our Resilience (CORE) [29], resilience-based intervention (RBI) [35,37], and resilience-based group intervention (RBGI) [36]. The Internet-based CORE intervention was conducted over a period of 6 weeks with the aim of teaching stress-coping skills and strategies, increasing resilience and well-being, and promoting self-empowerment [29]. CORE consisted of six interactive modules weekly sessions. The module contained psychoeducation, autonomy, increasing self-care and compassion, overcoming obstacles, building relationships with others, and life goals and personal growth. This intervention was shown to be effective in increasing resilience and skills as well as preventing common mental health disorders.

The RBI intervention consisted of twelve 1 h sessions [35]. The basic framework for this intervention was interactive discussion, free play, behavioral exercises, and self-regulation exercises. The topics in the interactive discussion were leadership, personal space, starting and maintaining conversations, and stress management. In addition, there were weekly assignments for students to practice the skills taught and record in achievement journals while at home. Another RBI intervention was carried out for 18 sessions with a time of 40 min per session [37]. The intervention was carried out in a hybrid manner by providing knowledge about programs regarding mental well-being in adolescents by increasing resilience. These interventions can improve resilience, intrapersonal relationships, and promote positive mental well-being.

The RBGI intervention aimed to build and increase resilience in students who are victims of bullying by building positive relationships between students and parents, engaging in positive activities, and building positive coping [36]. This program consisted of eight sessions using social media as a step to build inspiration into the implementation of activities. The activities also consisted of discussions and presentations related to bullying, self-reflection exercises, improving interpersonal relationships, increasing resilience, making peace with the past, and improving adaptive coping. After being given the intervention, students significantly increased their resilience and adaptive coping, and the incidence of bullying of students decreased.

The results of the study were analyzed by all authors and manually extracted into the table to facilitate the analysis of the results of the scoping review. The results of the analysis of the articles are presented in tabular form as follows (Table 2):

## 4. Discussion

The scoping review showed that nurses have an important role in reducing the incidence of bullying and its impact on students in schools. However, nurses cannot function alone, and they need support from parents and schools. In providing interventions, nurses focus on improving the health status of patients. Nurses provide nursing interventions using several methods, namely, preventive, promotive, curative, and rehabilitative actions [24]. Curative action to reduce the incidence of bullying and its impact can be achieved using physical therapy and peer support groups [38]. Another method is in the form of psychological therapy, which increases the ability of the patient to deal with the stressors from bullying received by students [39]. There are four types of nursing interventions that can be provided to reduce the incidence of bullying and its impact, namely, prevention programs, activities programs, peer group programs, and resilience programs.

The results of the study show that nursing interventions were administered to students at school. Most of the studies show that interventions were provided to students in high school. Nursing interventions to reduce the incidence of bullying and its impact must be tailored to the characteristics of students based on their developmental stage [40]. The effectiveness of nursing interventions on students in high schools is mostly achieved because students can be managed to reduce the impact of bullying in schools [41]. This is in contrast to students at university who already have a busy life and are independent in determining the activities to be carried out [42], while students at elementary school are still at the stage in childhood where they need guidance in carrying out their lives [43,44]. The results of the study show that the prevention program is an attempt to reduce the incidence of bullying and its impact on students in schools with several interventions, namely, a social cognitive theory (SCT)-based intervention, the Bullying Prevention Program (BPP), and a pragmatic school-based universal intervention. All three interventions focused on health education. The method to reduce the impact of bullying through health education was proven to be effective because there was a decrease in the incidence of bullying in schools [45]. Another study showed that health education with posters in schools can increase student awareness of the negative impact of bullying [46]. In addition, previous research showed that empathy training in schools can increase caring among students thereby reducing bullying behavior in schools [47,48].

Activity-focused interventions are actions implemented to reduce the impact of bullying, such as stress and traumatic experiences. A previous study showed that arts and sports activities were proven to reduce the incidence of bullying because students used their time for activities rather than bullying [49,50]. In addition, sports activities are also proven to improve mental health as students are more concerned about their friends [51].

The peer group intervention program is one of the therapies provided in groups. Each group was provided with the opportunity to discuss and share experiences about bullying [52]. The results of the review show that through the discussion and sharing of experiences between students, student awareness can increase [53]. In addition, the students who participated in peer groups felt they had a supportive environment at school [43]. This is in line with previous research which showed that students felt supported to learn after involvement in a peer group [54].

The results of the study indicate that the intervention of increasing student resilience reduces the impact of bullying. Resilience enhancement can be provided online and offline with interventions in the form of increasing self-esteem, setting goals, and increasing student motivation. This is in line with previous studies which showed that high student resilience can reduce the impact of bullying [55]. In addition, students will be resilient in the face of bullying and only focus on their goals at school.

The results of the study indicate that interventions can be carried out in a span of 8 weeks–6 months. This is in line with previous studies which showed that interventions to prevent and reduce bullying in students were effective for 3 months [56]. Another study showed that collaborative interventions involving nurses, schools, families, and students can be carried out within a minimum of 4 months [57]. A simple intervention can be implemented by providing health education to students to prevent the bullying of students at school [58].

Nursing interventions against bullying cannot only be implemented by nurses but require the involvement of many parties including other health workers, families, and schools. The prevention of bullying is the responsibility of all parties related to students in schools [59]. Family support is important in reducing the incidence of bullying because students are at home for a long time [60].

The provision of nursing interventions to students to prevent and reduce the impact of bullying needs to pay attention to the condition of students. The backgrounds of students need to be studied so that bullying can be handled appropriately [61]. Implementing inappropriate interventions can cause trauma in students [62,63]. Nursing interventions provided to students to prevent and reduce the impact of bullying will be effective if the correct intervention is implemented by the correct instructor and health worker in a supportive environment, according to the background of the student [64,65,66].

### Limitations

The limitation of this study is that the articles reviewed were limited to the last 10 years. Although it aims to provide the latest interventions and types of bullying, this study would be more comprehensive if it had reviewed more previous articles. Another limitation in this study is that the type of intervention is limited to nursing interventions; it does not involve other health workers, and health services for bullying are less integrated. The article also only discusses the incidence of bullying at school, while bullying can occur in the family at home or in the community. The rigor in this study is based on the fact that: the authors eliminated articles based on predetermined criteria; reread the articles before their review and analysis; and conducted discussions with all authors to analyze the articles covered by the review. The authors also read about the impact of nursing interventions in terms of their overall benefit in reducing the incidence of bullying and its impact on students.

## 5. Conclusions

Nursing interventions can reduce the impact and incidence of bullying on students at school. There are four types of nursing interventions that can be carried out, namely, prevention programs, activities programs, peer group programs, and resilience programs. These interventions can be implemented face-to-face, online, and as a hybrid. In addition to reducing the incidence of bullying and its impact on students at school, nursing interventions can also improve mental health in the form of self-efficacy, resilience, social skills, and adaptive coping. The implication of this study is that there is a methodological basis for nurses in the provision of nursing interventions to reduce the incidence of bullying and its impact on students in schools. Another implication is that schools can consider activities that involve health workers, including nurses, in order to reduce the incidence of bullying and its impact in schools. The results of the study can be used as a basis for future research on the effectiveness of resilience programs through nursing interventions to reduce the incidence of bullying and its impact on students in schools.

## Figures and Tables

**Table 1:** JBI critical appraisal tool.

Author, Published Year	JBI Critical Appraisal Tool	Study Design
[20]	76.9% (10/13)	RCT
[28]	92.3% (12/13)	RCT
[29]	76.9% (10/13)	RCT
[30]	76.9% (10/13)	RCT
[31]	92.3% (12/13)	RCT
[32]	88.9% (8/9)	Quasi-experiment
[33]	76.9% (10/13)	RCT
[34]	92.3% (12/13)	RCT
[35]	100% (13/13)	RCT
[36]	N/A	Mixed methods
[37]	88.9% (8/9)	RCT

**Table 2:** Extraction Data.

No	Author and Year	Purpose	Country	Method	Sample	Intervention	Result
1.	[20]	Effect of intervention based on social cognitive theory (SCT) on the incidence of bullying in schools	Iran	RCT	280 students at elementary school	Social cognitive theory (SCT)-based intervention	Interventions significantly reduce the incidence of bullying and victimization and improve the social skills of students at school
2.	[28]	Effectiveness in preventing and overcoming bullying of students in schools	United Kingdom	RCT	3214 students at university	Bullying Prevention Program	Effective interventions to prevent and overcome the impact of victimization on students in schools
3.	[29]	Effects of an Internet-based intervention (CORE: Cultivating our Resilience) on increasing resilience and well-being and reducing symptoms of depression and anxiety in students	Spain	Study protocol for RCT	464 students at high school	Internet-based intervention (CORE: Cultivating our Resilience)	This program is effective in improving resilience and coping skills in students and can reduce anxiety and depression in students
4.	[30]	Effect of universal school-based interventions on increasing resilience and reducing mental health problems in students	Australia	RCT	315 students at high school	Pragmatic school-based universal intervention	There is a significant effect, increasing resilience and decreasing mental health problems in students
5.	[31]	Effect of karate-based intervention on student resilience	Italy	RCT	50 students at elementary school	Shotokan Karate	The intervention is effective in increasing the resilience and well-being of students in schools
6.	[32]	Effectiveness of peer tutoring-based programs against the incidence of bullying and cyberbullying in schools	Spain	Quasi- experimental	2057 students at university	Tutoría Entre Iguales Program (school-based intervention of peer tutoring)	The results showed that there was a significant reduction in bullying, peer victimization, and cyberbullying behavior among students at school
7.	[33]	Effect of martial arts-based psychosocial intervention on increasing the resilience and self-efficacy of students	Australia	RCT	283 students at high school	Martial arts based-intervention	The results showed that there was an increase in the welfare, resilience, and self-efficacy of students at school
8.	[34]	Effect of the Learning Together intervention in developing the social and emotional skills of students in school	London	RCT	7121 students at university	Learning Together intervention.	This intervention has a small but significant effect on bullying, and there is an increase in the social and emotional abilities of students at school
9.	[35]	Assess the resilience builder program (RBP) in terms of improving student emotional regulation in response to bullying incidents at school	Texas	RCT	67 students at high school	Resilience-based intervention	RBP intervention has a significant impact on improving the emotional control of students in dealing with bullying incidents in school
10.	[36]	Effect of the resilience-based group intervention (RBGI) on the level of resilience in students at school	Philippines	Mixed-methods	72 students at high school	Resilience-based group intervention	The results showed that there was a significant increase in student resilience and adaptive coping at school
11.	[37]	Effect of resilience- based intervention on increasing resilience in students at school	Spain	RCT	6000 students at university	Resilience-based intervention.	The results showed that there was an increase in the capacity of resilience and adaptive coping abilities.

## Data Availability

Not applicable.
